# Effectiveness of CPR in Hypogravity Conditions—A Systematic Review

**DOI:** 10.3390/life12121958

**Published:** 2022-11-23

**Authors:** Remco Overbeek, Jan Schmitz, Lucas Rehnberg, Yacine Benyoucef, Fabian Dusse, Thais Russomano, Jochen Hinkelbein

**Affiliations:** 1 Department of Anesthesiology and Intensive Care Medicine, Faculty of Medicine and University Hospital Cologne, University of Cologne, 50937 Cologne, Germany; 2 German Society of Aerospace Medicine (DGLRM), 80331 Munich, Germany; 3 Space Medicine Group, European Society of Aerospace Medicine (ESAM), 51149 Cologne, Germany; 4 General Intensive Care Unit, University Hospital Southampton NHS Foundation Trust, Southampton SO16 6YD, UK; 5 InnovaSpace, London SE28 0LZ, UK; 6 Spacemedex, Valbonne Sophia-Antipolis, 06560 Valbonne, France; 7 Department of Physiatry and Nursing, Faculty of Health Sciences, IIS Aragon, University of Zaragoza, 50009 Zaragoza, Spain; 8 Department of Anesthesiology, Intensive Care Medicine and Emergency Medicine, Johannes-Wesling-Universitätsklinikum Minden, Ruhr-Universität Bochum, 32429 Minden, Germany

**Keywords:** cardiopulmonary resuscitation, hypogravity, basic life support

## Abstract

(1) Background: Cardiopulmonary resuscitation (CPR), as a form of basic life support, is critical for maintaining cardiac and cerebral perfusion during cardiac arrest, a medical condition with high expected mortality. Current guidelines emphasize the importance of rapid recognition and prompt initiation of high-quality CPR, including appropriate cardiac compression depth and rate. As space agencies plan missions to the Moon or even to explore Mars, the duration of missions will increase and with it the chance of life-threatening conditions requiring CPR. The objective of this review was to examine the effectiveness and feasibility of chest compressions as part of CPR following current terrestrial guidelines under hypogravity conditions such as those encountered on planetary or lunar surfaces; (2) Methods: A systematic literature search was conducted by two independent reviewers (PubMed, Cochrane Register of Controlled Trials, ResearchGate, National Aeronautics and Space Administration (NASA)). Only controlled trials conducting CPR following guidelines from 2010 and after with advised compression depths of 50 mm and above were included; (3) Results: Four different publications were identified. All studies examined CPR feasibility in 0.38 G simulating the gravitational force on Mars. Two studies also simulated hypogravity on the Moon with a force of 0.17 G/0,16 G. All CPR protocols consisted of chest compressions only without ventilation. A compression rate above 100/s could be maintained in all studies and hypogravity conditions. Two studies showed a significant reduction of compression depth in 0.38 G (−7.2 mm/−8.71 mm) and 0.17 G (−12.6 mm/−9.85 mm), respectively, with nearly similar heart rates, compared to 1 G conditions. In the other two studies, participants with higher body weight could maintain a nearly adequate mean depth while effort measured by heart rate (+23/+13.85 bpm) and VO_2max_ (+5.4 mL·kg^−1^·min^−1^) increased significantly; (4) Conclusions: Adequate CPR quality in hypogravity can only be achieved under increased physical stress to compensate for functional weight loss. Without this extra effort, the depth of compression quickly falls below the guideline level, especially for light-weight rescuers. This means faster fatigue during resuscitation and the need for more frequent changes of the resuscitator than advised in terrestrial guidelines. Alternative techniques in the straddling position should be further investigated in hypogravity.

## 1. Introduction

Cardiopulmonary resuscitation (CPR) is a vital form of basic life support (BLS) and advanced life support (ALS) during a cardiac arrest, as it maintains circulation and, therefore, cerebral perfusion and enhances the likelihood of survival [1]. On Earth, CPR has been studied extensively where international guidelines are reviewed and updated every five years [2]. However, this is not the case for CPR in altered gravitational conditions, such as microgravity and hypogravity.

With the growth of commercial space flight and planned crewed expeditions to the Moon and Mars, the probability of emergencies requiring CPR in space will increase [3]. Possible causes for cardiac arrest on Earth, such as the reversible causes outlined in resuscitation guidelines (‘4 Hs’ and ‘4 Ts’), are also possible in space, as well as on planetary and lunar surfaces [4]. The sources of these are maybe very different, such as decompression sickness from rapid decompression when going on an extra-vehicular activity (EVA), toxin exposure from an ammonia leak, or radiation illness from galactic cosmic radiation [5,6]. However, estimating the risk in microgravity is difficult, but it is likely, in a healthy population such as astronauts, that trauma leading to hemorrhage or pneumothorax, or possibly sepsis from infection, will be the most likely cause of cardiac arrest [7]. The populations are likely vastly different with a highly skilled, highly trained, and aerobically fit crew members who have also been extensively screened for significant comorbidities compared to a deconditioned patient in a hospital setting. Despite some deconditioning over a long-duration mission to Mars, they are likely to have a greater physiological reserve compared to most inpatients in a hospital setting, but their access to medical resources, postresuscitation, or critical care will be limited [3,4].

An expedition to Mars would overall take an expected 2.4 years and a crew of approximately seven members. Considering the incidence rate for a life-threatening medical emergency to be about 0.06 events per astronaut and year, that would lead to one estimated emergency per mission, which possibly will require CPR [8]. To date, there have been approximately 600 people that have flown to space and no recorded cardiac arrests. This reflects the benefit of astronaut selection, training, screening, and effective countermeasures, but does also mean that there is no outcome data from cardiac arrests in these environments.

While CPR under terrestrial conditions has been very well studied since the late 1950s and early 1960s [9], the implementation of this well-established technique under altered gravity conditions can be problematic. Current terrestrial guidelines emphasize the importance of appropriate depth (>50 mm) and rate (100–120 min^−1^) of external chest compressions [2]. The challenge of performing traditional terrestrial CPR under reduced gravity conditions is the ability to apply sufficient force to achieve the required depth of compression. This is crucial to provide clinically relevant compressions, give an adequate cardiac output to perfuse vital organs, and, finally, achieve the return of spontaneous circulation (ROSC). In microgravity, these problems are more apparent and lead to the development of, so far, seven different chest compression techniques [10,11], such as the Evetts–Russomano method [12], the Reverse Bear Hug method, or the Handstand method [13], which make CPR possible in the total absence of gravity or microgravity. These techniques have been summarized in the microgravity CPR guidelines [14]. In hypogravity, however, these techniques are not feasible, as there is still a gravitational field present, but significantly reduced compared to Earth’s. This means that CPR in the hypogravity conditions of the Moon and Mars follow terrestrial guidelines and techniques but are potentially less effective.

The aim of this systematic review is to determine if, by using current CPR guidelines, adequate chest compressions as part of CPR are feasible in hypogravity simulations on Earth.

## 2. Materials and Methods

### 2.1. Search Strategy

A systematic literature search was conducted in July 2021 by two independent reviewers. The databases of PubMed and the Cochrane Register of Controlled Trials, as well as the websites of ResearchGate and NASA, were searched for eligible studies. A search algorithm was developed to identify all relevant studies using Boolean operators.

Search Algorithm: (CPR OR Resuscitation OR Reanimation) AND (Hypogravity OR Mars OR Moon)

### 2.2. Eligibility Criteria and Data Collection

Prior to screening, inclusion and exclusion criteria were developed. Only controlled trials conducting chest compressions following guidelines from 2010 and after with advised compression depths of 50 mm and above were included in this review. For better comparison, hypogravity had to be simulated by body-suspension devices only. Parabolic flights were not considered for this review because hypogravity can only be simulated for 20 to 25 s, thus leaving no time for examining fatigue and a possible decrease of CPR quality. To evaluate the feasibility of CPR studies, we had to report on CPR effectiveness (compression depths/rate) as well as participant’s effort (heart rate/VO_2max_/Ve/Borg Score) [15].

**Figure 1:**
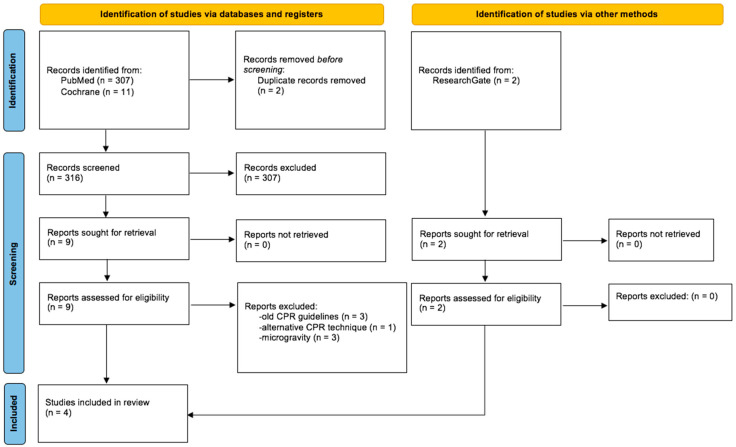
PRISMA flow diagram, adapted under the terms of the Creative Commons Attribution License from ‘The PRISMA 2020 statement: An updated guideline for reporting systematic reviews’ [16], 2021, Page, M.J.

After identification, all relevant publications were collated to Endnote X7 and screened following the updated 2020 PRISMA statement guideline [16] (Figure 1). Relevant data, including participant characteristics (sample size, weight, age), setting, CPR protocol, and outcome were extracted to Excel and tabulated. Three authors were contacted for missing data.

## 3. Results

A total of 320 records were identified. After removing duplicates and screening of title and abstract by two independent researchers, 11 full-text articles were assessed for eligibility while 307 articles were excluded, as the title and abstract did not match the topic. Seven studies had to be excluded for various reasons, thus leaving four studies to be included in this review (Figure 1).

### 3.1. Study Characteristics

All four studies had a Repeated Measures Design with volunteers being their own control group. Three trials were conducted at the Microgravity Center of the Pontifical Catholic University of Rio Grande do Sul in Brazil [17,18,19], and one in the Centre for Human and Applied Physiological Sciences of the King’s College in London [20].

Ninety-one participants with an average age and weight of 24.0 years and 76.8 kg, respectively, were included in the trials (Table 1). Most participants were university students who were screened with a questionnaire for any major health concerns, which would have prevented them from performing in the study.

**Figure 2:**
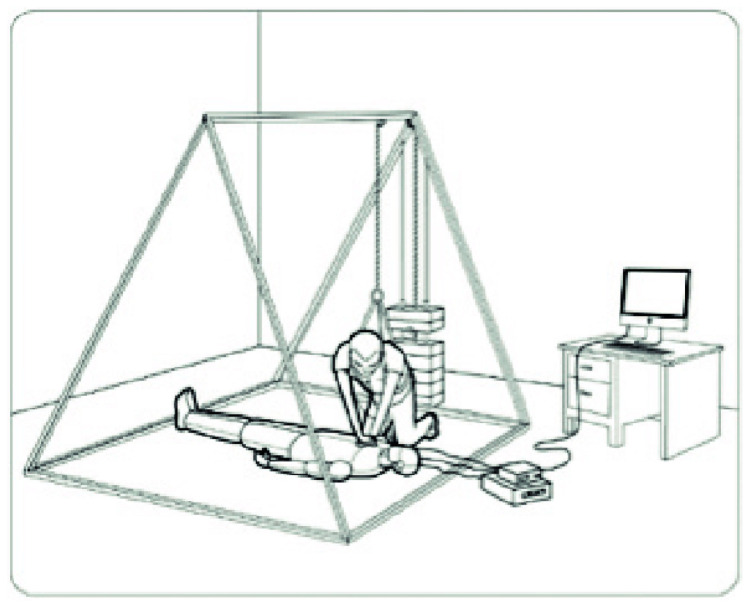
Body-suspension device [17,18,19], reprinted with permission from ‘Extraterrestrial CPR and Its Applications in Terrestrial Medicine’ [21]. 2010, Russomano T.

All studies simulated hypogravity with custom-built body-suspension devices consisting of a counterweight system connected to the participant via a body harness (Figure 2). To calculate the counterweight needed to simulate body mass at different hypogravity levels, all authors used similar equations: Counterweight = 0.6 × Body mass–(0.6 × body mass × Simulated gravitational force/1 G) All studies examined CPR feasibility in 0.38 G simulating the gravitational force on Mars. Two studies also simulated hypogravity on the Moon with a force of 0.17 G or 0.16 G (1 G = 9.81 m/s^2^) [17,20].

CPR was performed on standard CPR mannequins (e.g., Resusci Anne Skill Reporter, Laerdal Medical Ltd., Orpington, UK), which were modified to allow for measurement of compression depth and rate. Guidelines and CPR protocols can be seen in Table 2. Participants were given feedback on CPR quality with LED lights indicating compression depths [17,18,19], a metronome set to an advised compression rate of 100/s [17,18], or verbal feedback by study investigators [19,20].

### 3.2. Risk of Bias

The setting and design of the studies made certain quality criteria such as the blinding of the participants or allocation impossible. Based on the Cochrane Handbook [22], four categories were created to assess the risk of bias (Table 3).

(1) Selection Bias: Do participants represent possible astronauts concerning variability of for example gender, age, or weight?
(2) Order Bias: Is the order of gravity conditions randomized to eliminate influence of fatigue or learning effect?
(3) Intervention Bias: Do setting and surroundings of intervention adequately resemble an emergency in hypogravity?
(4) Reporting Bias: Is there any selective reporting or missing data?

### 3.3. CPR Quality

CPR quality was assessed by measurement of compression depth and rate (Table 4). One study [17] reported True-Depth, calculated by subtracting inadequate recoil of maximum depth. It had been noted by the authors in this study, and some microgravity studies, that participants would not always fully release the chest, thereby not allowing the chest to fully recoil before the next chest compression. The ‘true depth’ was devised to allow a more accurate measure of depth of these chest compressions [18].

A compression rate above 100/s could be maintained in all studies and hypogravity conditions. Baptista et al. [19] only reported a nonsignificant difference without actual numbers. In comparison between 1 G and 0.38 G, the mean depth achieved during compressions was reduced in all trials. Only one study could maintain a compression depth above 50 mm and therefore meet the required guideline depth [18]. In 0.17 G, both studies failed to fulfil CPR guidelines by more than 5 mm [17,20].

### 3.4. Physical Effort

All studies recorded the participant’s heart rate throughout the resuscitation. Two studies showed a significantly higher heart rate after CPR in Martian conditions than in terrestrial control [18,19]. The other two showed no difference between Martian, terrestrial, or lunar conditions (Table 5) [17,20].

**Table 5:** Post-CPR heart rate (1/min); * after 90 s.

Publication	1 G	Post-Heart Rate 0.38 G	0.17 G/0.16 G
Baptista	107.43 (17.12)	121.29 (27.03)	-----
Mackaill	130 (11.4)	127 (13.9)	130 (20.3)
Russomano	117 (21)	140 (21)	------
Sriharan *	106.18 (14.71)	108.97 (20.53)	108.54 (19.34)

Russomano et al. showed that, with higher heart rates in hypogravity, participants also had significantly higher VO_2_ and minute ventilation (Ve) [18]. In the study of Sriharan et al. [20], neither heart rate nor VO_2_ were significantly higher in Martian or lunar conditions (Table 6). Three studies assessed Borg scores and recorded an increase in perceived exertion in hypogravity (Table 7) [18,19,20].

**Table 6:** VO2peak (mL/kgmin)/Ve (L/min); * after 1 min.

Publication	1 G	VO2peak 0.38 G	0.17 G/0.16 G	1 G	Ve 0.38 G	0.17 G/0.16 G
Russomano	16.4 (4.5)	21.8 (8.1)	-----	27.5 (7.9)	40.6 (10.2)	-----
Sriharan *	14.4 (7.8)	16.8 (7.2)	15.9 (6.2)	20.6 (11.7)	23.7 (10.6)	22.9 (8.8)

**Table 7:** Borg: * estimated from graph; ** after 5 min.

Publication	1 G	BORG 0.38 G	0.17 G/0.16 G
Baptista	9.33 (2.29)	12.42 (1.78)	---
Russomano *	10.2	13.2	
Sriharan **	10.5 (2.7)	11.4 (2.4)	11.9 (2.8)

### 3.5. Elbow Flexion

Elbow flexion was measured, as it enables the rescuer to compensate for the loss of body weight by recruiting upper arm muscles. In two studies, the flexion of the elbow was measured and it was found that the reduction of the gravitational force increased the flexion accordingly (Table 8).

## 4. Discussion

Future space tourists and astronauts will experience long exposure to microgravity when travelling to extraterrestrial environments. Cardiovascular alterations during these spaceflights can potentially harm astronauts both in-flight was well as after reaching the surface [23]. Astronauts show decreases in plasma volume, anemia, muscle atrophy, and fluid shifts, which lead to decreases in circulating blood volume and can affect cardiac output [24]. This has been shown in flight as well as in ground analogues [25].

There have been numerous cases of arrhythmias in space during the Apollo era (1961–1972) as well as the Skylab missions (1973–1979). The Russian Federation reported 75 cases of arrhythmias during the MIR era (1986–2001) [26]. During long-term spaceflight, astronauts will be exposed to cosmic radiation, which affects the cardiovascular system by triggering endothelial dysfunction and increasing aortic stiffness [5]. A total of 83% of astronauts returning from long-duration flights showed symptoms of orthostatic intolerance, which poses several hazards, including being able to carry out mission critical tasks and responding to emergencies when they arrive on other planets, such as performing CPR in a cardiac arrest scenario [27]. Future astronauts need to be prepared to handle medical emergencies autonomously, which includes adequate BLS and CPR, which is vital in cases of cardiac arrest to ensure survival.

Initial studies examining the feasibility of classic CPR in hypogravity conditions started in 2006 at the Aerospace Engineering Laboratory of the Microgravity Center-PUCRS in Brazil, first using the BSD to simulate reduced gravitational fields [28]. First results following old AHA 2000 guidelines [29] showed that classic CPR was feasible in lunar and Martian conditions, but compression depths above 40 mm could not always be fulfilled by participants with low body weight [28]. In a study by Kordi et al. in 2012 [30], a compression depth above 40 mm could be maintained by all participants, but female rescuers performed significantly worse than their male counterparts. In the study of Krygiel et al. [31], an only male group could maintain 2005 compression standards with significantly increased physical effort (heart rate +40% compared to 1 G). Since 2010, the recommended compression depth in resuscitation guidelines has been increased from 40–50 mm to 50–60 mm. This means an increased effort for the resuscitator, especially in conditions of reduced gravity, and raises the question whether CPR can be adequately performed in these conditions, especially for low-weight resuscitators [29].

In the trials of Russomano et al. [18] and Baptista et al. [19], only male participants were included. Compression depths and rates did not differ significantly from 1 G to 0,38 G. Physiological costs, however, measured subjectively with the Borg scale (Table 7) and objectively with VO2 (Table 6) and heart rate (Table 5) increased. The results indicate a sufficient aerobic reserve of males to achieve adequate BLS for the period of testing.

Moreover, the range of elbow flexion increased, indicating recruitment of the upper arm muscles (Table 8). This contradicts traditional CPR instructions, where the use of straight and rigid arms is advised to perform compressions. In hypogravity, however, the participants needed to generate more force by flexing end extending the upper limbs to compensate for the loss of body weight. Kaminska et al. [32] showed that trunk muscle mass and left and right arm muscle mass were positively correlated with compression depth in terrestrial conditions. Consequently, muscle mass may play an even more important role in compensating for weight loss and the prevention of being pushed away from the patient in hypogravity. As anthropometric data from the general population [33], as well as former astronauts [34], show that females have less body weight and a smaller muscle mass than males, this would explain a possible gender difference in CPR quality in hypogravity.

Sriharan et al. [20] and Mackaill et al. [17] conducted CPR in Martian and lunar conditions. Unlike the other trials, Sriharan et al. included female participants. Both studies included resuscitators with significantly less body weight (Table 1). The compression depth decreased according to the decreasing level of gravity and did not reach the guideline values in any hypogravity condition. This reduction in CPR quality would reduce organ perfusion and decrease chances of survival in actual cases of emergency [35]. Perceived exertion and post-CPR heart rates, however, did not increase significantly, leaving the question whether CPR quality could have been higher with more physical effort. Only in the study of Sriharan et al. CPR was performed for a longer period (5 min). For better comparison, 90 s values were retrieved; however, as initiation of Advanced Life support (ALS) can take as long as 4 min during a space mission, BLS and CPR have to be administered longer than tested in most of the trials. As compression depth tends to decay after 90 s [36], effective compressions will not be possible for that duration in hypogravity. The data from Sriharan et al. confirmed this significant effect of time of CPR on compression depth. Consequently, in environments with reduced gravitational fields, there may need to be a higher rate of change of the resuscitator than advised in terrestrial guidelines.

**Figure 3:**
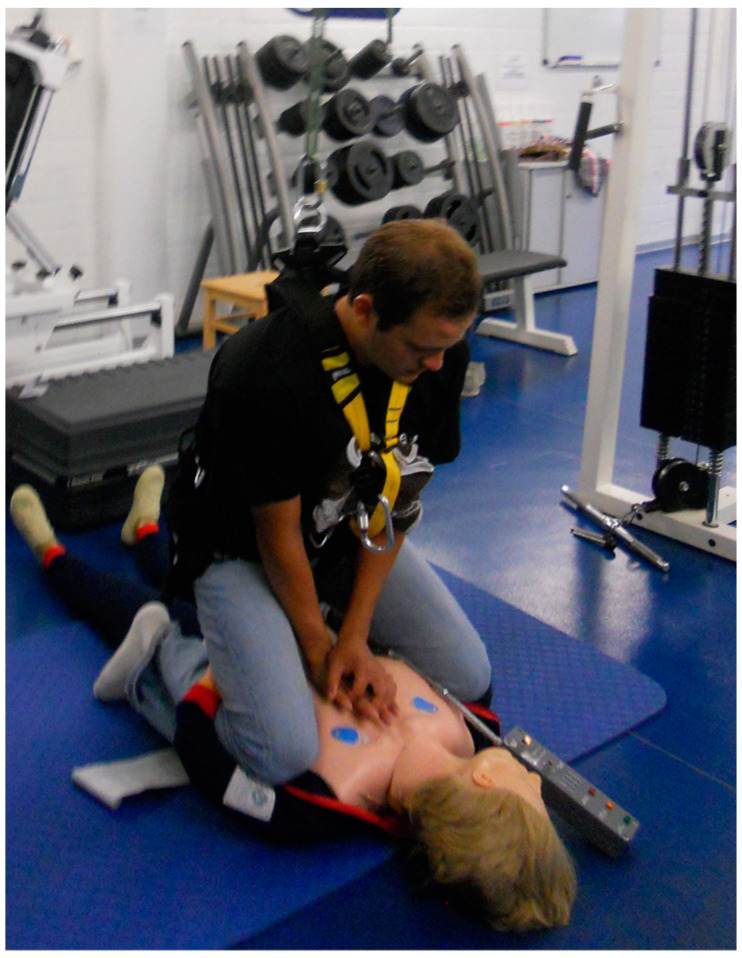
The Seated-Arm-Lock method [37].

The results show that adequate CPR quality cannot always be guaranteed when performing classic CPR following terrestrial guidelines in hypogravity conditions. Similar to the techniques developed for microgravity, there could be a benefit in adjusting to the reduced effective body weight by stabilizing the resuscitator and therefore preventing being pushed away from the patient. So far, two similar techniques in straddling position, the Mackaill–Russomano [17] and the Seated-Arm-Lock method [37] (Figure 3) have been developed. The rescuer is stabilized by either locking the victim’s arms behind the rescuer’s knees or tucking the rescuer’s heels and lower legs underneath the subject’s legs (Mackaill–Russomano). As no significant differences are found in the quality of chest compressions or the rescuers’ comfort and fatigue levels using the straddle position in 1 G [38], this might be a possibility to enhance CPR quality in hypogravity.

The studies showed that a body-suspension device can successfully simulate different hypogravity levels. In contrast to parabolic flights (25 s to 32 s time of hypogravity), CPR can be administered for a longer duration of time, which allows to record for fatigue and physical effort. Nevertheless, hypogravity ground simulations have limitations. Reduced aerobic capacity and muscular deconditioning after long-term microgravity exposure can hardly be reproduced on Earth [39]. LED lights and metronomes used in the simulation as audio–visual feedback of depth and rate may not be available. Chest-wall compliance of mannequins, as well as chest-wall dynamics in hypogravity, are not considered. All trial simulated scenarios are only possible inside of planetary bases. During possible missions outside of planetary bases, astronauts would have to wear EVA suits, which would make adequate CPR significantly harder due to a rising metabolic cost [40]. Based on current EVA suits used aboard the International Space Station (ISS), and some of the proposed future designs, the stiffness of the victim’s suit might not allow for adequate compression depth; in that case, protocols for rapid doffing would have to be implemented.

When BLS protocols are implemented for hypogravity conditions, the use of mechanical devices such as LUCAS, Autopulse, or Corpuls CPR has to be considered. Although there is no clear evidence of a benefit of these devices on Earth in terms of survival [41], they could be of value on exploration-class missions and in hypogravity, as fatigue is a more important factor, as is team- and resource-management.

## 5. Conclusions

With lower levels of gravity and reduced body weight, CPR becomes more physically demanding. Whilst compression rates above 100/min can be maintained in lunar and Martian conditions, adequate compression depth can only be maintained by resuscitators with higher body weight. To compensate the reduction in weight, elbow flexion increases and upper limb muscles are recruited. Consequently, the recommendation for straight and rigid arms during resuscitation on Earth cannot apply for hypogravity conditions. As females tend to weigh less and have a smaller muscle mass, they are more likely to perform inadequate compressions. As future crews on exploration-class missions will be diverse, concerning gender, body shape, and size, it will influence CPR performance. A higher rate of change of the resuscitator than advised in terrestrial guidelines is recommended (<1 min). Future investigation is needed with male and female participants reflecting the diversity of future space crews enabling to assess the effects of gender, body mass, aerobic capacity, and muscle strength on CPR quality. Alternative techniques in straddling position such as the Mackaill–Russomano [17] or the Seated-Arm-Lock method [37] are likely to improve CPR quality and should be further examined. Astronauts should be trained in the most effective technique for microgravity, as well as hypogravity, as part of a BLS training in ground analogues, for example, with the body-suspension device.

## Figures and Tables

**Table 1:** Participant characteristics.

Publication	Participants	% Males/Females	Age (Years)	Weight (kg)
Baptista	30	100/0	22 (3.0)	78.9 (10.6)
Mackaill	10	---	24.5 (2,8)	74.2 (11.1)
Russomano	30	100/0	22.5 (3.5)	78.2 (13.1)
Sriharan	21	52.3/47.7	28.7 (5.7)	72.9 (16)

**Table 2:** Setting * LED lights green 50–60 mm; ** 100/s.

Publication	Guideline	Simulation	Protocol	Feedback CD	Feedback CR	Hypogravity
Baptista	AHA 2010	Body-Suspension Device	3 × 30c	Visual *	Verbal	0.38 G
Mackaill	ERC 2015	Body-Suspension Device	3 × 30c	Visual *	Metronome **	0.38 G + 0.17 G
Russomano	ERC 2010	Body-Suspension Device	4 × 30c	Visual *	Metronome **	0.38 G
Sriharan	ERC 2015	Body-Suspension Device	5 min	Verbal initial 20 s	Verbal initial 20 s	0.38 G + 0.16 G

**Table 3:** Risk of bias: x high–some concerns + low.

Publication	Selection Bias	Order Bias	Intervention Bias	Reporting Bias
Baptista	x	--	--	--
Mackaill	x	--	--	+
Russomano	x	+	--	+
Sriharan	--	+	+	+

**Table 4:** Compression depth (mm)/rate (1/min) * after 90 s; ** True-Depth; # no numbers reported.

Publication	1 G	Compression Depth 0.38 G	0.17 G/0.16 G	1 G	Compression Rate 0.38 G	0.17 G/0.16 G
Baptista	48.09 (2.61)	45.92 (2.84)	------	#	#	-----
Mackaill **	56.6 (2.0)	49.4 (3.4)	44.0 (3.5)	101.7	106.4	106.4
Russomano	57.0 (2.3)	55.1 (3.7)	------	104.25 (3.5)	103 (5.3)	------
Sriharan *	52.94 (10.48)	44.23 (9.98)	43.09 (9.55)	107.39 (6.59)	107.75 (6.75)	108.14 (7.41)

**Table 8:** Elbow flexion (°).

Publication	1 G	Elbow Flexion 0.38 G	0.17 G/0.16 G
Mackaill	3.8 (1.5)	6.5 (1.6)	8.6 (3.1)
Russomano	4.3 (2.8)	14.0 (8.1)	------

## Data Availability

Data is contained within the article.

## Abbreviations

CPR	Cardiopulmonary Resuscitation
AHA	American Heart Association
NASA	National Aeronautics and Space Administration
VO2peak	Peak Oxygen Consumption
Ve	Minute Ventilation
BLS	Basic Life Support
ALS	Advanced Life Support
SeAL	Seated-Arm-Lock
MR	Mackaill–Russomano
ROSC	Return of Spontaneous Circulation
EVA	Extravehicular Activity
EMG	Electromyography
CD	Compression Depth
CR	Compression Rate
BSD	Body-Suspension Device

## References

[1] Sasson C., Rogers M.F., Dahl J., Kellermann A.L. (2010). Predictors of survival from out-of-hospital cardiac arrest: A systematic review and meta-analysis. Circ. Cardiovasc. Qual. Outcomes.

[2] Merchant R.M., Panchal A.R., Cheng A., Aziz K., Berg K.M., Lavonas E.J., Magid D.J. (2020). Part 1: Executive Summary: 2020 American Heart Association Guidelines for Cardiopulmonary Resuscitation and Emergency Cardiovascular Care. Circulation.

[3] Komorowski M., Fleming S., Mawkin M., Hinkelbein J. (2018). Anaesthesia in austere environments: Literature review and considerations for future space exploration missions. NPJ Microgravity.

[4] Hinkelbein J., Russomano T., Hinkelbein F., Komorowski M. (2018). Cardiac arrest during space missions: Specificities and challenges. Trends Anaesth. Crit. Care.

[5] Boerma M., Nelson G.A., Sridharan V., Mao X.W., Koturbash I., Hauer-Jensen M. (2015). Space radiation and cardiovascular disease risk. World J. Cardiol..

[6] Hodkinson P.D., Anderton R.A., Posselt B.N., Fong K.J. (2017). An overview of space medicine. Br. J. Anaesth..

[7] Antonsen E.A.-O., Myers J.A.-O., Boley L., Arellano J., Kerstman E., Kadwa B., Buckland D.M., Van Baalen M. (2022). Estimating medical risk in human spaceflight. NPJ Microgravity.

[8] Summers R.L., Johnston S.L., Marshburn T.H., Williams D.R. (2005). Emergencies in Space. Ann. Emerg. Med..

[9] Criley J.M., Niemann J.T., Rosborough J.P. (1984). Cardiopulmonary resuscitation research 1960–1984: Discoveries and advances. Ann. Emerg. Med..

[10] Braunecker S., Douglas B., Hinkelbein J. (2015). Comparison of different techniques for in microgravity—A simple mathematic estimation of cardiopulmonary resuscitation quality for space environment. Am. J. Emerg. Med..

[11] Schmitz J., Ahlbäck A., DuCanto J., Kerkhoff S., Komorowski M., Löw V., Russomano T., Starck C., Thierry S., Warnecke T. (2022). Randomized Comparison of Two New Methods for Chest Compressions during CPR in Microgravity-A Manikin Study. J. Clin. Med..

[12] Evetts S.N., Evetts L.M., Russomano T., Castro J.C., Ernsting J. (2005). Basic life support in microgravity: Evaluation of a novel method during parabolic flight. Aviat. Space Environ. Med..

[13] Jay G.D., Lee P., Goldsmith H., Battat J., Maurer J., Suner S. (2003). CPR effectiveness in microgravity: Comparison of three positions and a mechanical device. Aviat. Space Environ. Med..

[14] Hinkelbein J., Kerkhoff S., Adler C., Ahlbäck A., Braunecker S., Burgard D., Cirillo F., De Robertis E., Glaser E., Haidl T.M. (2020). Cardiopulmonary resuscitation (CPR) during spaceflight—A guideline for CPR in microgravity from the German Society of Aerospace Medicine (DGLRM) and the European Society of Aerospace Medicine Space Medicine Group (ESAM-SMG). Scand. J. Trauma Resusc. Emerg. Med..

[15] Borg G. (1970). Perceived exertion as an indicator of somatic stress. Scand J. Rehabil. Med..

[16] Page M.J., McKenzie J.E., Bossuyt P.M., Boutron I., Hoffmann T.C., Mulrow C.D., Shamseer L., Tetzlaff J.M., Akl E.A., Brennan S.E. (2021). The PRISMA 2020 statement: An updated guideline for reporting systematic reviews. Syst. Rev..

[17] Mackaill C., Sponchiado G., Leite A.K., Dias P., Da Rosa M., Brown E.J., de Lima J.C.M., Rehnberg L., Russomano T. (2018). A new method for the performance of external chest compressions during hypogravity simulation. Life Sci. Space Res..

[18] Russomano T., Baers J.H., Velho R., Cardoso R.B., Ashcroft A., Rehnberg L., Gehrke R.D., Dias M.K.P., Baptista R.R. (2013). A comparison between the 2010 and 2005 basic life support guidelines during simulated hypogravity and microgravity. Extrem. Physiol. Med..

[19] Baptista R., Susin T., Dias M., Corria N., Cardoso R., Russomano T. (2015). Muscle Activity during the Performance of CPR in Simulated Microgravity and Hypogravity. Am. J. Med. Biol. Res..

[20] Sriharan S., Kay G., Lee J.C.Y., Pollock R.D., Russomano T. (2021). Cardiopulmonary Resuscitation in Hypogravity Simulation. Aerosp. Med. Hum. Perform..

[21] Russomano T., Rehnberg L., Aslanidis T. (2017). Extraterrestrial CPR and Its Applications in Terrestrial Medicine. Resuscitation Aspects, [Internet].

[22] Higgins J.P.T., Chandler J., Cumpston M., Li T., Page M.J., Welch V.A. (2021). Cochrane Handbook for Systematic Reviews of Interventions, version 6.2 (updated February 2021); Cochrane, London, UK. http://www.training.cochrane.org/handbook.

[23] Sides M.B., Vernikos J., Convertino V.A., Stepanek J., Tripp L.D., Draeger J., Hargens A.R., Kourtidou-Papadeli C., Pavy-LeTraon A., Russomano T. (2005). The Bellagio Report: Cardiovascular risks of spaceflight: Implications for the future of space travel. Aviat. Space Environ. Med..

[24] West J.B. (2000). Physiology in microgravity. J. Appl. Physiol..

[25] Tanaka K., Nishimura N., Kawai Y. (2017). Adaptation to microgravity, deconditioning, and countermeasures. J. Physiol. Sci..

[26] Vernice N.A.-O., Meydan C., Afshinnekoo E., Mason C.E. (2020). Long-term spaceflight and the cardiovascular system. Precis Clin. Med..

[27] Meck J.V., Reyes C.J., Perez S.A., Goldberger A.L., Ziegler M.G. (2001). Marked exacerbation of orthostatic intolerance after long- vs. short-duration spaceflight in veteran astronauts. Psychosom. Med..

[28] Dalmarco G., Calder A., Falcão F., de Azevedo D.F., Sarkar S., Evetts S., Moniz S., Russomano T. (2006). Evaluation of external cardiac massage performance during hypogravity simulation. Conf. Proc. Annu. Int. Conf. IEEE Eng. Med. Biol. Soc..

[29] Panchal A.R., Bartos J.A., Cabañas J.G., Donnino M.W., Drennan I.R., Hirsch K.G., Kudenchuk P.J., Kurz M.C., Lavonas E.J., Morley P.T. (2020). Part 3: Adult Basic and Advanced Life Support: 2020 American Heart Association Guidelines for Cardiopulmonary Resuscitation and Emergency Cardiovascular Care. Circulation.

[30] Kordi M., Kluge N., Kloeckner M., Russomano T. (2012). Gender influence on the performance of chest compressions in simulated hypogravity and microgravity. Aviat. Space Environ. Med..

[31] Krygiel R.G., Waye A.B., Baptista R.R., Heidner G.S., Rehnberg L., Russomano T. (2014). The evaluation of upper body muscle activity during the performance of external chest compressions in simulated hypogravity. Life Sci. Space Res..

[32] Kaminska H., Wieczorek W., Matusik P., Czyzewski L., Ladny J.R., Smereka J., Filipiak K.J., Szarpak L. (2018). Factors influencing high-quality chest compressions during cardiopulmonary resuscitation scenario, according to 2015 American Heart Association Guidelines. Kardiol. Pol..

[33] Miller A.E., Tarnopolsky M.A., Sale D.G. (1993). Gender differences in strength and muscle fiber characteristics. Eur. J. Appl. Physiol. Occup. Physiol..

[34] Harm D.L., Jennings R.T., Meck J.V., Powell M.R., Putcha L., Sams C.P., Schneider S.M., Shackelford L.C., Smith S.M., Whitson P.A. (2001). Genome and Hormones: Gender Differences in Physiology Invited Review: Gender issues related to spaceflight: A NASA perspective. J. Appl. Physiol..

[35] Lurie K.G., Nemergut E.C., Yannopoulos D., Sweeney M. (2016). The Physiology of Cardiopulmonary Resuscitation. Anesth. Analg..

[36] Sugerman N.T., Edelson D.P., Leary M., Weidman E.K., Herzberg D.L., Vanden Hoek T.L., Becker L.B., Abella B.S. (2009). Rescuer fatigue during actual in-hospital cardiopulmonary resuscitation with audiovisual feedback: A prospective multicenter study. Resuscitation.

[37] Benyoucef Y., Keady T., Marwaha N. (2014). The Seated Arm-Lock Method: A New Concept of Basic Life Support in Simulated Hypogravity of the Moon and Mars. J. Space Saf. Eng..

[38] Supatanakij P., Yuksen C., Chantawong T., Sawangwong P., Jenpanitpong C., Patchkrua J., Kanchayawong P. (2020). Straddle versus Conventional Chest Compressions in a Confined Space; a Comparative Study. Arch. Acad. Emerg. Med..

[39] Demontis G.C., Germani M.M., Caiani E.G., Barravecchia I., Passino C., Angeloni D. (2017). Human Pathophysiological Adaptations to the Space Environment. Front. Physiol..

[40] Norcross J.R., Clark T., Harvill L., Morency R.M., Stroud L.C., Desantis L., Vos J.R., Gernhardt M.L. (2010). Metabolic Costs and Biomechanics of Level Ambulation in a Planetary Suit.

[41] Bonnes J.L., Brouwer M.A., Navarese E.P., Verhaert D.V., Verheugt F.W., Smeets J.L., de Boer M.-J. (2016). Manual Cardiopulmonary Resuscitation Versus CPR Including a Mechanical Chest Compression Device in Out-of-Hospital Cardiac Arrest: A Comprehensive Meta-analysis from Randomized and Observational Studies. Ann. Emerg. Med..

